# Asphericity of tumor FDG uptake in non-small cell lung cancer: reproducibility and implications for harmonization in multicenter studies

**DOI:** 10.1186/s13550-020-00725-y

**Published:** 2020-11-02

**Authors:** Julian M. M. Rogasch, Christian Furth, Stephanie Bluemel, Piotr Radojewski, Holger Amthauer, Frank Hofheinz

**Affiliations:** 1 Department of Nuclear Medicine, Charité – Universitätsmedizin Berlin, corporate member of Freie Universität Berlin, Humboldt-Universität Zu Berlin, and Berlin Institute of Health, Augustenburger Platz 1, 13353 Berlin, Germany; 2 Institute for Radiopharmaceutical Cancer Research, Helmholtz-Zentrum Dresden-Rossendorf, Dresden, Germany

**Keywords:** FDG-PET, Image reconstruction, Spatial resolution, Asphericity, Non-small cell lung cancer, Reproducibility, Prognosis

## Abstract

**Background:**

Asphericity (ASP) of the primary tumor’s metabolic tumor volume (MTV) in FDG-PET/CT is independently predictive for survival in patients with non-small cell lung cancer (NSCLC). However, comparability between PET systems may be limited. Therefore, reproducibility of ASP was evaluated at varying image reconstruction and acquisition times to assess feasibility of ASP assessment in multicenter studies.

**Methods:**

This is a retrospective study of 50 patients with NSCLC (female 20; median age 69 years) undergoing pretherapeutic FDG-PET/CT (median 3.7 MBq/kg; 180 s/bed position). Reconstruction used OSEM with TOF_4/16_ (iterations 4; subsets 16; in-plane filter 2.0, 6.4 or 9.5 mm), TOF_4/8_ (4 it; 8 ss; filter 2.0/6.0/9.5 mm), PSF + TOF_2/17_ (2 it; 17 ss; filter 2.0/7.0/10.0 mm) or Bayesian-penalized likelihood (Q.Clear; beta, 600/1750/4000). Resulting reconstructed spatial resolution (FWHM) was determined from hot sphere inserts of a NEMA IEC phantom. Data with approx. 5-mm FWHM were retrospectively smoothed to achieve 7-mm FWHM. List mode data were rebinned for acquisition times of 120/90/60 s. Threshold-based delineation of primary tumor MTV was followed by evaluation of relative ASP/SUVmax/MTV differences between datasets and resulting proportions of discordantly classified cases.

**Results:**

Reconstructed resolution for narrow/medium/wide in-plane filter (or low/medium/high beta) was approx. 5/7/9 mm FWHM. Comparing different pairs of reconstructed resolution between TOF_4/8_, PSF + TOF_2/17_, Q.Clear and the reference algorithm TOF_4/16_, ASP differences was lowest at FWHM of 7 versus 7 mm. Proportions of discordant cases (ASP > 19.5% vs. ≤ 19.5%) were also lowest at 7 mm (TOF_4/8_, 2%; PSF + TOF_2/17_, 4%; Q.Clear, 10%). Smoothing of 5-mm data to 7-mm FWHM significantly reduced discordant cases (TOF_4/8_, 38% reduced to 2%; PSF + TOF_2/17_, 12% to 4%; Q.Clear, 10% to 6%), resulting in proportions comparable to original 7-mm data. Shorter acquisition time only increased proportions of discordant cases at < 90 s.

**Conclusions:**

ASP differences were mainly determined by reconstructed spatial resolution, and multicenter studies should aim at comparable FWHM (e.g., 7 mm; determined by in-plane filter width). This reduces discordant cases (high vs. low ASP) to an acceptable proportion for TOF and PSF + TOF of < 5% (Q.Clear: 10%). Data with better resolution (i.e., lower FWHM) could be retrospectively smoothed to the desired FWHM, resulting in a comparable number of discordant cases.

## Background

Patients with early-stage or locally advanced non-small cell lung cancer (NSCLC) are potential candidates for curatively intended therapy; however, management decisions are primarily based on the clinical tumor stage as a single factor only [1]. In the average of patients, adjuvant chemotherapy only showed modest survival benefits [2–4], and therefore, more effective methods of treatment selection are highly warranted.

Consequently, numerous additional prognostic or predictive factors [5–7], among image-derived parameters [8–12], have been investigated aiming at more differentiated outcome prediction and more differentiated management decisions. Among parameters from positron emission tomography/computed tomography with [^18^F]fluorodeoxyglucose (FDG-PET/CT), asphericity (ASP) is a parameter that reflects shape irregularity of the primary tumor’s metabolic tumor volume (MTV), combining metric and metabolic features of the primary tumor. Three retrospective studies confirmed its independent prognostic value for progression-free (PFS) and overall survival (OS) in patients with NSCLC [13–15]. The largest study (311 patients, UICC stage I–III) further showed that ASP, with a cutoff of > 19.5%, could identify patients with UICC stage II treated by surgery and adjuvant chemotherapy with high ASP and reduced PFS (median 11 months vs. not reached) and OS (22 months vs. not reached) [15]. ASP was superior for survival prediction compared to primary tumor’s maximum standardized uptake value (SUVmax) and MTV, two other previously proposed and common PET parameters [8, 9, 16, 17].

Studies on quantitative PET parameters have mostly been monocentric, but the main limitation of any PET parameter is its dependence on numerous technical factors including image reconstruction algorithms. Therefore, results may fail to reproduce in a multicenter approach unless harmonization between centers is ensured [18–20]. SUVmax and MTV may vary by > 30% if basic ordered subset expectation maximization (OSEM) reconstruction is combined with time-of-flight (TOF) information and/or scanner-specific compensation for the point spread function (PSF) [19–22].

Variability of ASP has not been investigated so far, but an impact of different reconstruction methods and resulting levels of image noise can be expected. The definition of ASP includes the MTV and its surface; therefore, a variability of MTV will cause variability of ASP. Since MTV also varies notably depending on the applied delineation algorithm [20, 23–25], there are two potential sources of variability of ASP: image generation and lesion delineation.

The goal of the current study was to investigate differences in ASP resulting from variability in image generation (common reconstruction methods and acquisition times). The focus was on the assessment if the resulting variation is acceptable for application in multicenter studies and on defining the range of acceptable variation of the influencing factors. Specifically, the goal was not to investigate the trueness of ASP itself, to identify a ground truth or to define a highly optimized reconstruction protocol for a specific PET scanner. To the contrary, this study investigated whether ASP could still be used in multicenter studies under imperfect clinical conditions with different scanners and a certain variation in acquisition protocols (uptake time, acquisition time). Such variability introduced by image generation should be separated from variations in image post-processing, the software for image feature extraction [26] or variation in lesion delineation. Therefore, data were not post-processed (unless specified), and the same software and delineation method were used as in the preceding studies on ASP in NSCLC [13–15]. To facilitate interpretation, SUVmax and MTV were investigated analogously for comparison.

## Methods

### Phantom data

A NEMA IEC body phantom was examined using a GE Discovery MI PET scanner (GE Healthcare, General Electric, Boston, MA, USA) with a 3-ring detector with silicon photomultipliers (SiPM) and a reported sensitivity of 7.3 cps/kBq [27]. Total activity in field of view was approximately 35 MBq. The absolute activities were measured in a certified dose calibrator (ISOMED 2010, MED Dresden GmbH, Germany), which was also used for regular cross calibration of the PET scanner (every 6 months). Sphere inserts (inner diameter 10, 13, 17, 22, 28, and 37 mm) were filled with 24.4 kBq/ml F18-fluoride, while the background was filled with 3.1 kBq/ml (sphere-to-background ratio, approx. 8:1). Acquisition time was 3 min per bed position (transaxial field of view, 70 cm; matrix size, 256 × 256; voxel size, 2.73 × 2.73 × 2.78 mm^3^). CT data of the phantom were used for attenuation correction. Scatter correction, random correction and dead time correction were also performed.

PET raw data were reconstructed using OSEM with time of flight (TOF; GE “VUE Point FX”) with 4 iterations and 16 subsets (i.e., TOF_4/16_). This reconstruction was defined as the reference algorithm for subsequent analyses and used either a 2.0-mm, 6.4-mm or 9.5-mm in-plane Gaussian filter (i.e., TOF_4/16/2_, TOF_4/16/6.4_ or TOF_4/16/9.5_). Further reconstruction was performed with OSEM and TOF with 4 iterations, 8 subsets and either 2.0 mm, 6.0 mm or 9.5 mm in-plane filter (TOF_4/8/2_, TOF_4/8/6_ or TOF_4/8/9.5_).

Additionally, data were reconstructed using OSEM with TOF and point spread function (OSEM + PSF + TOF, hereafter referred to as PSF + TOF; GE “VUE Point FX” with “SharpIR”) with 2 iterations and 17 subsets and either 2.0-mm, 7.0-mm or 10.0-mm in-plane filter (PSF + TOF_2/17/2_, PSF + TOF_2/17/7_ or PSF + TOF_2/17/10_), respectively. TOF and PSF + TOF reconstructions always included a “standard” z-axis filter.

All data were also reconstructed using Bayesian-penalized likelihood reconstruction (GE “Q.Clear”) with a penalization factor β of 600, 1750 or 4000 (Q.Clear_600_, Q.Clear_1750_ or Q.Clear_4000_), respectively.

**Fig. 1:**
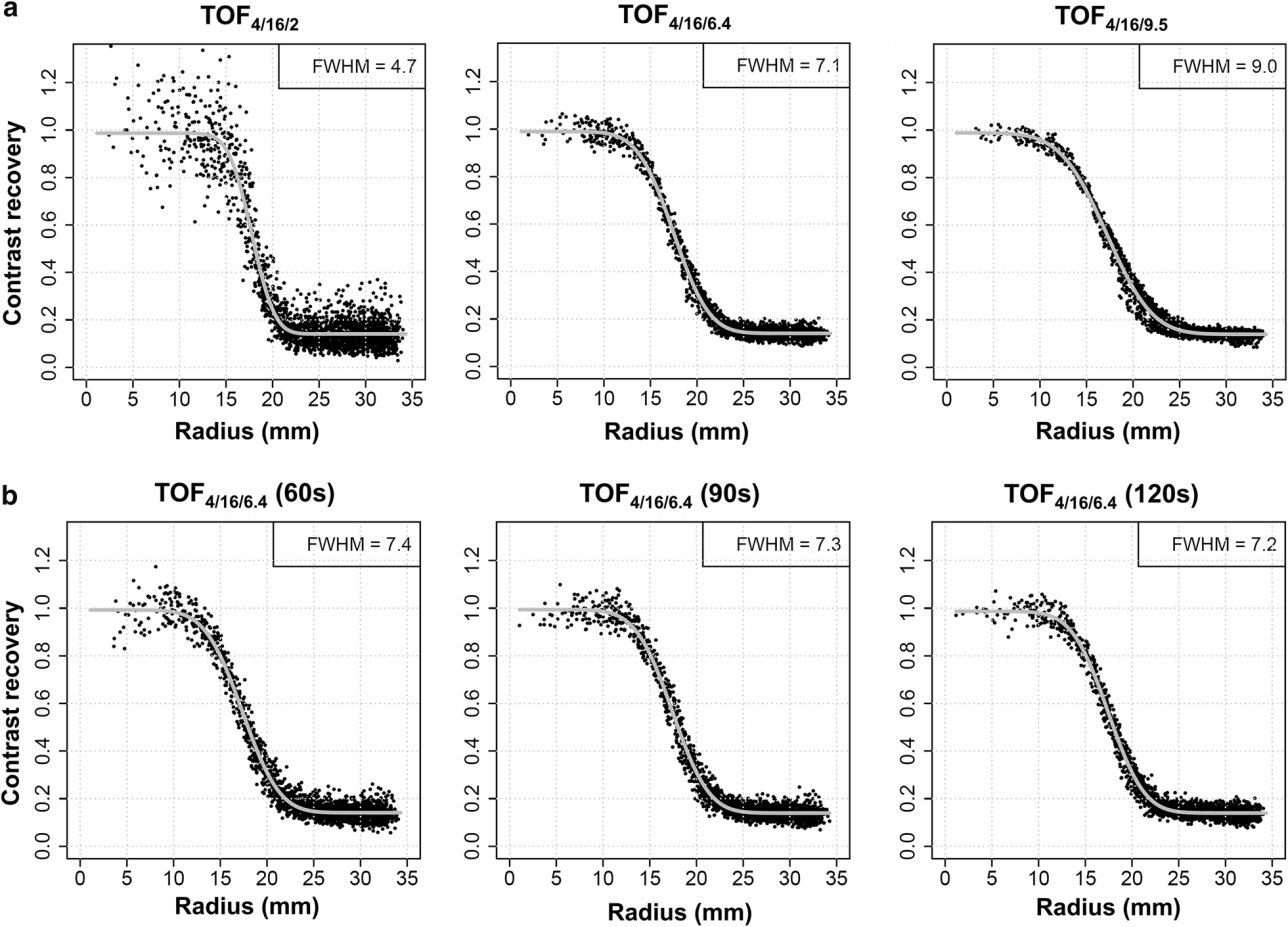
Sphere activity profiles. **a** Radial activity profiles of the 37-mm sphere for the reference algorithm with different in-plane filter widths to achieve different levels of reconstructed spatial resolution (FWHM). Acquisition time was 180 s. Substantial noise propagation can be observed at FWHM of approx. 5 mm. **b** Corresponding profiles for 6.4-mm in-plane filter width at shorter acquisition times. Noise especially increases between 90 and 60 s acquisition time, while reconstructed spatial resolution remains similar

Reconstructed spatial resolution was assessed as the full width at half maximum (FWHM) of the PSF in the reconstructed phantom images. PSF was modeled by a 3D Gaussian, and FWHM was determined by applying the method described in detail by Hofheinz et al*.* [28]. This method is based on fitting the analytic solution for the radial activity profile of a homogeneous sphere convolved with a 3D Gaussian to the reconstructed data. In this process, the full 3D vicinity of each sphere is evaluated by transforming the data to spherical coordinates relative to the respective sphere's center. A summary of the used reconstructions, resulting spatial resolution and image noise (patient data) is given in Table 1. Representative radial profiles are shown in Fig. 1.

**Table 1:** Reconstruction parameters and image noise

Spatial resolution	Parameters	Noise, % (mean ± SD)
5 mm		
TOF_4/8/2_	4 it, 8 ss, 2.0 mm	20.6 ± 4.4
TOF_4/16/2_	4 it, 16 ss, 2.0 mm	33.2 ± 7.1
PSF + TOF_2/17/2_	2 it, 17 ss, 2.0 mm	12.1 ± 2.5
Q.Clear_600_	beta = 600	6.9 ± 1.3
7 mm		
TOF_4/8/6_	4 it, 8 ss, 6.0 mm	8.1 ± 1.8
TOF_4/16/6.4_	4 it, 16 ss, 6.4 mm	10.1 ± 2.4
PSF + TOF_2/17/7_	2 it, 17 ss, 7.0 mm	6.8 ± 1.6
Q.Clear_1750_	beta = 1750	4.1 ± 0.9
9 mm		
TOF_4/8/9.5_	4 it, 8 ss, 9.5 mm	5.3 ± 1.2
TOF_4/16/9.5_	4 it, 16 ss, 9.5 mm	6.5 ± 1.5
PSF + TOF_2/17/10_	2 it, 17 ss, 10.0 mm	5.3 ± 1.3
Q.Clear_4000_	beta = 4000	3.0 ± 0.9

Reconstruction parameters for each reconstruction are displayed as well as resulting image noise in patient data (mean and standard deviation of all 50 patients)

it, iterations; ss, subsets; SD, standard deviation

To study effects of different acquisition time per bed position, PET list mode data were retrospectively rebinned to reconstruct further datasets representing an acquisition time of 120 s, 90 s or 60 s, respectively. Reconstruction was then performed with the algorithms that resulted in a reconstructed spatial resolution of 7 mm (i.e., TOF_4/8/6_, TOF_4/16/6.4_, PSF + TOF_2/17/7_ and Q.Clear_1750_).

### Patients and scans

Fifty patients (female 20; median age 69 years; range 46 to 83 years) with histologically proven NSCLC underwent pretherapeutic FDG-PET/CT between July 2018 and February 2019 using the same scanner. Patients were required to fast for at least 6 h prior to tracer administration, and a blood glucose level of ≤ 150 mg/dl was ensured. A median activity of 249 MBq (interquartile range [IQR], 238 to 257 MBq; range 209 to 274 MBq) or 3.7 MBq/kg (IQR 3.1 to 4.2 MBq/kg; range 2.0 to 5.7 MBq/kg) was administered intravenously. Static PET data were acquired after a median uptake time of 65 min (IQR 61 to 70 min; range 55 to 96 min) from the base of skull to the proximal femora in 3D acquisition mode (acquisition time, 180 s per bed position; bed overlap, approx. 25%). Attenuation correction was based on a non-enhanced low-dose CT (automated tube current modulation “Smart mA”; maximum tube current–time product 100 mAs; tube voltage 120 kV; gantry rotation time 0.5 s) or non-enhanced diagnostic CT (maximum tube current–time product, 200 mAs).

**Fig. 2:**
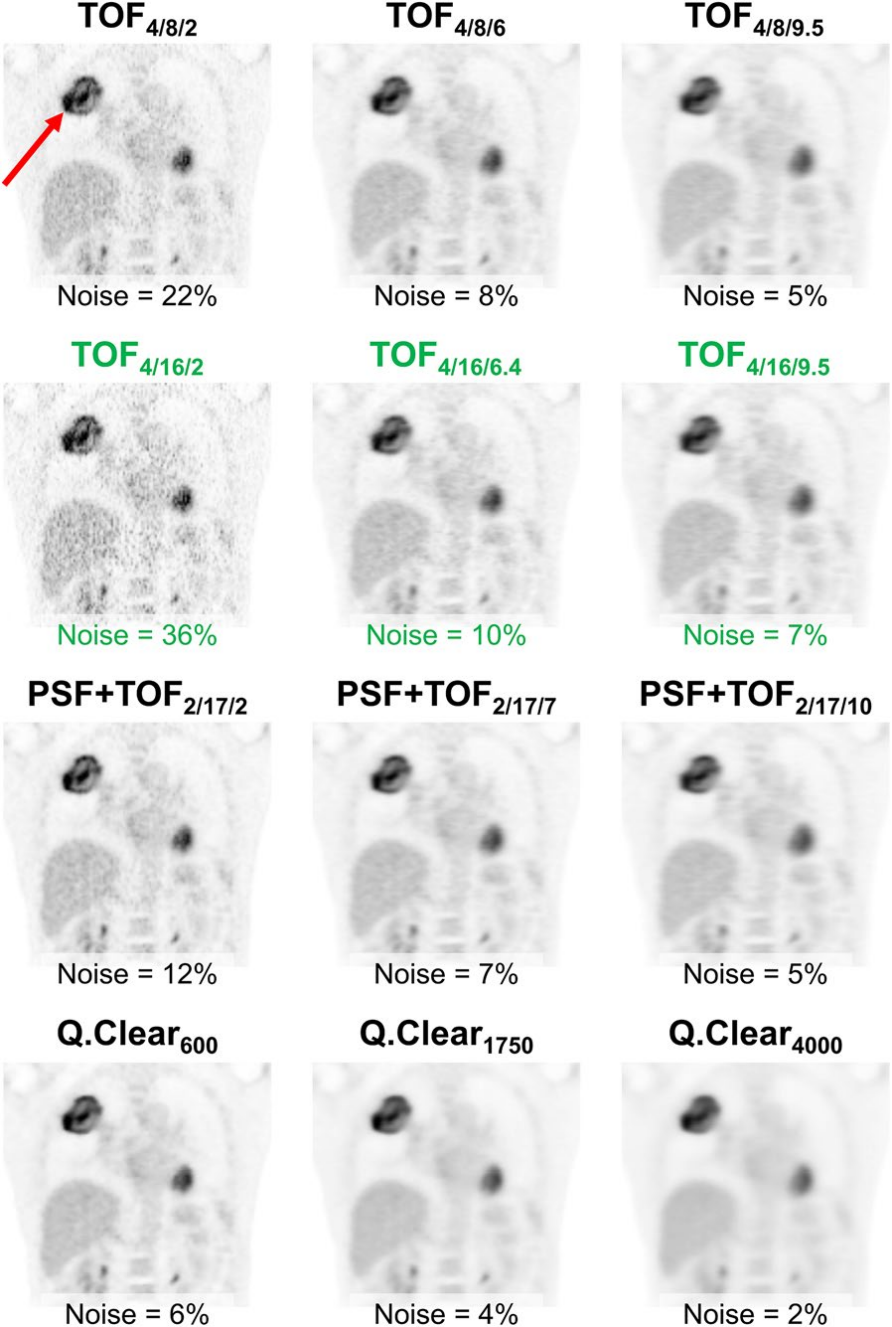
Patient example. Coronar FDG-PET images of the thorax for a patient are displayed for all 12 reconstruction algorithms (body mass index 22.5 kg/m^2^; injected activity 3.5 MBq/kg; acquisition time 180 s per bed position). The given noise level is the median of all 50 patients. Data are separated by reconstructed spatial resolution of approx. 5 mm (left column), 7 mm (middle column) or 9 mm FWHM (right column), respectively. The reference algorithm is highlighted in green. At 7 mm FWHM spatial resolution, ASP of the primary tumor (red arrow) was concordantly high (> 19.5%) with all algorithms except for Q.Clear

PET raw data were reconstructed as described above (patient example in Fig. 2). Furthermore, data with 5-mm FWHM resolution were smoothed with a Gaussian filter (5 mm FWHM). According to

1 $${\text{FWHM}}_{{{\text{target}}}}^{2} = {\text{FWHM}}_{{{\text{original}}}}^{2} + {\text{FWHM}}_{{{\text{filter}}}}^{2}$$

this results in a target spatial resolution of approximately 7 mm. Altogether, 25 image data per patient with different spatial resolution and noise (i.e., acquisition time) were generated.

### Data evaluation

Evaluation of the data was performed with a dedicated software (ROVER, version 3.0.34, ABX advanced biochemical compounds GmbH, Radeberg, Germany) by an experienced physician in nuclear medicine. MTV of the primary tumor was delineated in each dataset using the same threshold-based, background-adapted algorithm [29]. Delineation was visually inspected and manually corrected if deemed necessary. Tumoral FDG-avid tissue not related to the primary tumor and delineable from the latter (lymph nodes, metastases) was excluded. If the primary tumor was determined to be multifocal (i.e., separate ipsilateral tumor nodules) or the presence of lymphangitic carcinomatosis was diagnosed by interdisciplinary consensus, all tumor nodules and FDG-avid lymphangitic tissue were included in the MTV (see also [15]). SUVmax and ASP [30] of the MTV were derived. SUV was normalized using the body weight in kg.

ASP was calculated identical to its initial definition by the authors [30], which was unaltered in subsequent publications [13–15, 31–37]:

2 $${\text{ASP}} \left( \% \right) = \left( {\sqrt[3]{H} - 1} \right)*100\% \quad {\text{with}}\quad H = \frac{1}{36\pi }*\frac{{S^{3} }}{{V^{2} }}$$

S and V are the surface area and the volume of the MTV, respectively. S was computed as the sum of all voxel surfaces that form the outer and inner surfaces of the MTV multiplied by the factor 2/3. Note that this corresponds to the approximation of the surface area of discrete 3D objects using six voxel classes as described by [38].

**Fig. 3:**
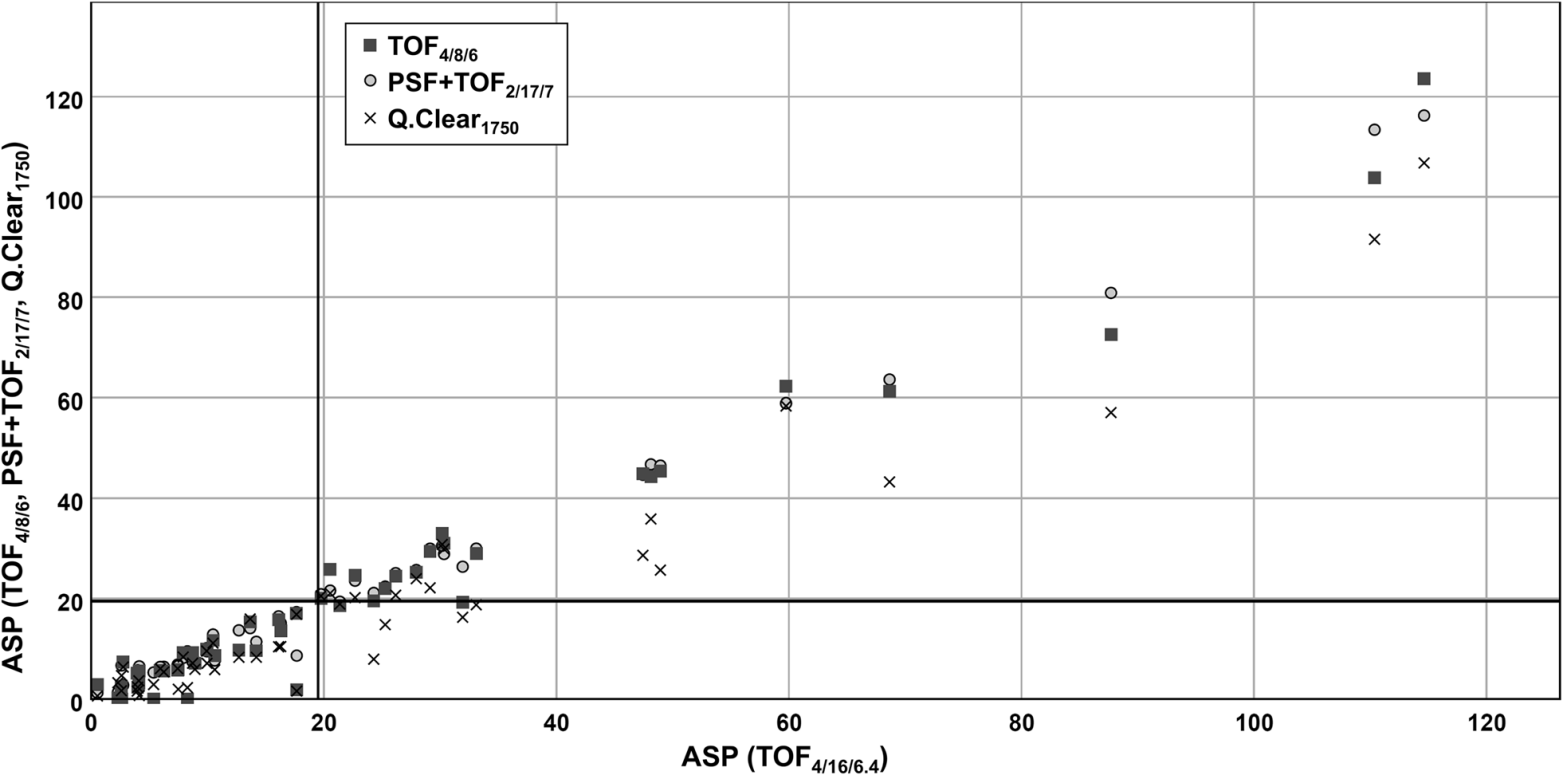
Distribution of ASP values with the reference algorithm. ASP values at reconstructed spatial resolution of 7.0-mm FWHM and acquisition time of 180 s are displayed for each of the 50 patients for TOF_4/8/6_, PSF + TOF_2/17/7_ and Q.Clear_1750_. The cutoff at 19.5% is highlighted at each axis. Several tumors feature ASP in proximity to this cutoff. Data points that are located either in the left upper section or in the right lower section of the diagram represent discordantly classified cases when compared to the reference algorithm TOF_4/16/6.4_ (TOF_4/8/6_, n = 1; PSF + TOF_2/17/7_, n = 2; Q.Clear_1750_, n = 5 discordant cases)

Please note that this definition of the MTV surface area is distinctly different from the definition by the Image Biomarker Standardization Initiative (IBSI), and compliance of both definitions cannot be assumed. The IBSI estimates the MTV surface area using a mesh-based representation after triangulation of the MTV’s outer surface [26]. Additional file 1 provides the IBSI checklist for an overview of all methodological aspects of image generation and image processing in the present analysis. Distribution of ASP values in all current 50 patients is illustrated in Fig. 3.

In each dataset, a spherical volume of interest (VOI) of approx. 19 ml was placed in the unaffected right liver lobe to derive its SUVmean and SUV standard deviation and calculate image noise (SUV standard deviation/SUVmean).

### Statistical analysis

Statistical analysis was performed using SPSS 22 (IBM Corporation, Armonk, NY, USA). Descriptive parameters were expressed as median and IQR. Relative differences between any dataset *a* and the reference dataset *b* were calculated as follows:

3 $${\text{Relative}}\,{\text{difference}}\,(\% ) = \frac{{\left| {a - b} \right|}}{b} \times 100\%$$

The significance of these differences was assessed with Wilcoxon signed-rank test for paired data. Proportions (%) of discordantly classified cases (high vs. low ASP/SUVmax/MTV) between algorithms were given with their 95% binomial proportion confidence intervals (95% CI), which included the continuity correction of ± 0.5/*n* (= ± 0.5/50 = ± 1%). Classification with ASP (> 19.5%) was based on a previously identified cutoff in NSCLC patients [15] while cutoffs for SUVmax (> 10.5) and MTV (> 9.5 ml) were the respective median among the current 50 patients. Proportions between different pairs of algorithms were compared with two-sided McNemar’s test. Correlation between ASP and MTV was examined using the Pearson correlation coefficient r and interpretation criteria based on [39]. Statistical significance was generally assumed at *p* < 0.05.

## Results

### Relative differences

To identify the level of reconstructed spatial resolution that provides minimal relative ASP difference to the reference algorithm (TOF_4/16_), different combinations of spatial resolution for candidate algorithms (TOF_4/8_, PSF + TOF_2/17_, Q.Clear) and the reference algorithm were compared pairwise (Table 2).

**Table 2:** Relative differences to the reference algorithm

	Relative differences, % (median, IQR)				
	5 mm versus 7 mm	7 mm versus 7 mm	9 mm versus 7 mm	5 mm versus 5 mm	9 mm versus 9 mm
ASP					
TOF_4/8_	205 (108–314)	7.6 (3.1–18.0)	38.8 (25.1–55.7)	30.7 (22.7–42.7)	9.3 (3.7–29.7)
TOF_4/16_	363 (187–603)	–	33.5 (23.2–58.9)	–	–
PSF + TOF_2/17_	85.3 (35.8–162)	12.8 (6.3–26.9)	34.4 (21.8–56.9)	50.1 (36.4–73.5)	16.2 (4.5–34.1)
Q.Clear	24.7 (15.4–51.4)	31.3 (11.2–43.7)	47.8 (26.2–63.9)	73.1 (54.1–85.1)	29.1 (9.1–44.6)
SUVmax					
TOF_4/8_	34.0 (25.7–41.3)	1.2 (0.6–2.0)	12.8 (10.2–17.2)	6.2 (3.8–10.4)	0.7 (0.4–2.0)
TOF_4/16_	43.1 (35.4–53.9)	–	12.6 (9.9–16.4)	–	–
PSF + TOF_2/17_	39.1 (28.6–46.2)	4.9 (1.9–7.2)	11.2 (7.5–13.9)	12.1 (3.3–20.7)	3.0 (1.6–5.1)
Q.Clear	17.2 (11.9–28.1)	5.1 (2.4–10.6)	11.7 (5.5–21.4)	14.6 (9.5–27.4)	7.1 (2.2–11.2)
MTV					
TOF_4/8_	29.9 (21.4–39.8)	2.3 (1.4–4.4)	11.7 (6.6–22.0)	6.2 (3.9–15.9)	1.3 (0.6–2.8)
TOF_4/16_	36.4 (24.9–50.7)	–	12.4 (6.4–22.4)	–	–
PSF + TOF_2/17_	31.9 (24.2–39.4)	6.1 (3.1–12.7)	9.3 (5.3–17.3)	21.7 (8.4–41.3)	3.4 (1.5–7.3)
Q.Clear	14.6 (7.6–20.7)	6.3 (3.1–13.2)	10.3 (4.0–17.2)	30.0 (13.2–83.7)	10.8 (5.9–16.3)

Relative differences in % (with interquartile range; IQR) are given for each algorithm relative to the reference algorithm TOF_4/16_. Different pairs of reconstructed spatial resolution (FWHM) are compared. Missing values reflect pairs of identical datasets

Relative ASP differences with TOF_4/8_ and PSF + TOF_2/17_ compared to TOF_4/16_ were significantly lower at 7 versus 7 mm than at 5 versus 7 mm, 9 versus 7 mm and 5 versus 5 mm (each *p* < 0.001). In contrast, differences with Q.Clear versus TOF_4/16_ at 7 versus 7 mm (median, 31.3%; IQR, 11.2 to 43.7%) were similar to 9 versus 7 mm (24.7%; 15.4 to 51.4%; *p* = 0.25). Relative ASP differences at 7 versus 7 mm were similar to 9 versus 9 mm with TOF_4/8_ (median, 7.6% vs. 9.3%; *p* = 0.38), PSF + TOF_2/17_ (12.8% vs. 16.2%; *p* = 0.25) and Q.Clear (31.3% vs. 29.1%; *p* = 0.33).

Relative SUVmax and MTV differences at 7 versus 7 mm were significantly lower than corresponding ASP differences (each *p* < 0.001; Table 2).

### Proportions of discordantly classified cases (original data)

The proportion of discordantly classified cases (ASP > 19.5% vs. ASP ≤ 19.5%) with TOF_4/8_ compared to the reference algorithm at 7 versus 7 mm was 2% (95% CI 0–6.9%) and significantly lower than at 5 versus 7 mm or 9 versus 7 mm (38% and 16%, each *p* < 0.05; Table 3) but similar to 5 versus 5 mm and 9 versus 9 mm (6% and 2%, each *p* > 0.5).

**Table 3:** Discordant cases relative to the reference algorithm (ASP)

	Discordant proportion, % (95% CI)				
	5 mm versus 7 mm	7 mm versus 7 mm	9 mm versus 7 mm	5 mm versus 5 mm	9 mm versus 9 mm
Strictly 19.5%					
TOF_4/8_	38 (23.5–52.5)	2 (0–6.9)	16 (4.8–27.2)	6 (0–13.6)	2 (0–6.9)
TOF_4/16_	44 (29.2–58.8)	–	14 (3.4–24.6)	–	–
PSF + TOF_2/17_	12 (2.0–22.0)	4 (0–10.4)	12 (2.0–22.0)	32 (18.1–45.9)	6 (0–13.6)
Q.Clear	10 (0.7–19.3)	10 (0.7–19.3)	26 (12.8–39.2)	38 (23.5–52.5)	12 (2.0–22.0)
5% tolerance					
TOF_4/8_	36 (21.7–50.3)	0 (0–1.0)	12 (2.0–22.0)	4 (0–10.4)	0 (0–1.0)
TOF_4/16_	36 (21.7–50.3)	–	10 (0.7–19.3)	–	–
PSF + TOF_2/17_	10 (0.7–19.3)	0 (0–1.0)	8 (0–16.5)	30 (16.3–43.7)	0 (0–1.0)
Q.Clear	10 (0.7–19.3)	6 (0–13.6)	22 (9.5–34.5)	36 (21.7–50.3)	10 (0.7–19.3)

Proportions of discordantly classified cases among all 50 patients are given in % (95%-confidence interval; 95% CI) for each algorithm relative to the reference algorithm TOF_4/16_. Different pairs of reconstructed spatial resolution (FWHM) are compared. Missing values reflect pairs of identical datasets. Proportions are provided either for a strict ASP cutoff (high, > 19.5%; low, ≤ 19.5%) or with 5% tolerance (i.e., ASP was also rated concordant if between 18.53% and 20.48%)

Conversely, PSF + TOF_2/17_ showed significantly lower proportions at 7 versus 7 mm (4%; 95% CI 0–10.4%) compared to 5 versus 5 mm (32%, *p* = 0.001), while proportions were similar to 5 versus 7 mm, 9 versus 7 mm and 9 versus 9 mm (12%, 12% and 6%, each *p* > 0.1).

Q.Clear resulted in significantly lower proportions of discordant cases at 7 versus 7 mm (10%; 95% CI 0.7–19.3%) than at 9 versus 7 mm and 5 versus 5 mm (26% and 38%, each *p* < 0.01), while proportions were similar to 5 versus 7 mm and 9 versus 9 mm (10% and 12%, each *p* = 1.0).

Proportions at 7 versus 7 mm were comparable between TOF_4/8_ and PSF + TOF_2/17_ (2% vs. 4%; *p* = 1.0), while both algorithms showed slightly less discordant cases than Q.Clear (10%; each *p* > 0.1).

Proportions of discordant cases at 7 versus 7 mm were comparable between ASP, SUVmax and MTV with TOF_4/8_ (2% vs. 6% vs. 2%; each *p* > 0.5), PSF + TOF_2/17_ (4% vs. 0% vs. 4%; each *p* = 1.0) and Q.Clear (10% vs. 6% vs. 8%; each *p* = 1.0; Additional file 2: Table S1).

The number of discordantly classified cases tended to decrease when allowing a ± 5% tolerance range around the ASP cutoff value (i.e., low ASP, < 20.48%; high ASP, > 18.53%; Table 3).

### Relative differences and discordant cases (retrospectively smoothed data)

Comparing data that were retrospectively smoothed to achieve 7-mm reconstructed spatial resolution with the original 7 mm data, relative differences between TOF_4/8_ and the reference algorithm TOF_4/16_ were higher in retrospectively smoothed data for ASP but similar for SUVmax and MTV (details in Table 4). In contrast, relative differences with PSF + TOF_2/17_ were comparable for ASP and significantly higher in the smoothed data for SUVmax and MTV. With Q.Clear, relative differences for ASP, SUVmax and MTV were each significantly lower in the smoothed data compared to original 7-mm data.

**Table 4:** Relative differences to the reference algorithm: smoothed data

	Relative differences, % (median, IQR)		*p* value
	Smoothed to 7 mm versus 7 mm	Original 7 mm versus 7 mm	
ASP			
TOF_4/8_	13.9 (9.3–32.8)	7.6 (3.1–18.0)	**0.001**
TOF_4/16_	9.1 (4.9–26.2)	–	–
PSF + TOF_2/17_	17.7 (5.2–37.6)	12.8 (6.3–26.9)	0.9
Q.Clear	17.6 (5.9–37.2)	31.3 (11.2–43.7)	**< 0.001**
SUVmax			
TOF_4/8_	1.6 (0.7–2.9)	1.2 (0.6–2.0)	0.08
TOF_4/16_	1.9 (1.0–3.3)	–	–
PSF + TOF_2/17_	8.9 (4.4–12.6)	4.9 (1.9–7.2)	**< 0.001**
Q.Clear	4.8 (1.8–6.6)	5.1 (2.4–10.6)	**0.021**
MTV			
TOF_4/8_	3.2 (1.5–5.8)	2.3 (1.4–4.4)	0.18
TOF_4/16_	3.1 (1.2–4.8)	–	–
PSF + TOF_2/17_	10.6 (5.4–16.5)	6.1 (3.1–12.7)	**< 0.001**
Q.Clear	6.2 (3.4–9.8)	6.3 (3.1–13.2)	**0.003**

Relative differences in % (with interquartile range; IQR) are given for each algorithm relative to the reference algorithm TOF_4/16_ at 7 mm FWHM (i.e., TOF_4/16/6.4_). Differences are displayed separately for either the retrospectively smoothed data (5 mm smoothed to 7 mm FWHM) or original 7 mm data. Missing values reflect pairs of identical datasets. Significant *p* values are printed in bold

Proportions of discordantly classified cases at 7 versus 7 mm were comparable between retrospectively smoothed data and original 7 mm data for TOF_4/8_ (smoothed vs. original, 2% vs. 2%; *p* = 1.0), for PSF + TOF_2/17_ (4% vs. 4%; *p* = 1.0) and Q.Clear (6% vs. 10%; *p* = 0.5). The rate of discordant cases between retrospectively smoothed data and original 7-mm data for the reference algorithm TOF_4/16_ itself was 2% (95% CI 0–6.9%).

### Relative differences and discordant cases (reduced acquisition time)

Relative differences in ASP, SUVmax and MTV at reconstructed spatial resolution of 7 mm (TOF_4/8/6_, TOF_4/16/6.4_, PSF + TOF_2/17/7_ and Q.Clear_1750_) and shorter acquisition times are displayed in Additional file 2: Tables S2 to S4. Independent from the acquisition time for the candidate algorithms, relative differences were always calculated with regard to the reference algorithm TOF_4/16/6.4_ at 180 s. Briefly, relative ASP, SUVmax and MTV differences with TOF_4/8/6_ and TOF_4/16/6.4_ were significantly higher at any shorter acquisition time (i.e., 120 s, 90 s and 60 s) than at 180 s. Relative differences with PSF_2/17/7_ tended to remain similar between 180 and 90 s but increased significantly at 60 s. Q.Clear_1750_ mostly showed similar ASP, SUVmax and MTV differences between all acquisition times.

Proportions of discordantly classified cases of ASP, SUVmax and MTV with TOF_4/8/6_, PSF + TOF_2/17/7_ and Q.Clear_1750_ did not increase significantly with shorter acquisition time (each compared to 180 s; Additional file 2: Tables S5 to S7). Discordant cases with TOF_4/16/6.4_ remained similar at 120 s and 90 s but increased with 60 s acquisition time (McNemar’s test not applicable).

### Correlation of ASP and MTV

**Fig. 4:**
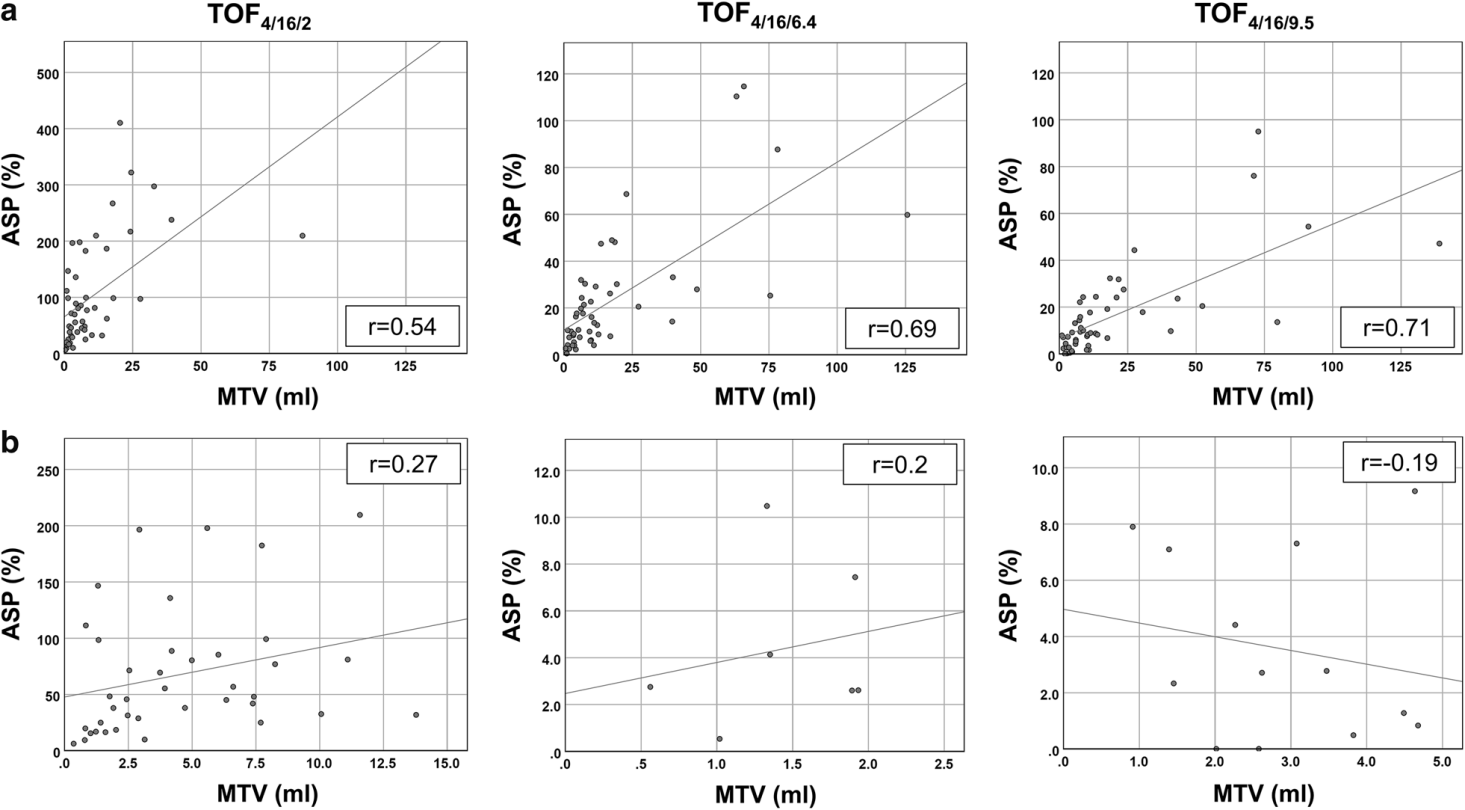
Correlation plots for ASP and MTV. Correlation plots for ASP and MTV for the three TOF_4/16_ algorithms. **a** shows plots for all patients. Correlation was moderate with TOF_4/16/2_ and moderate to high with TOF_4/16/6.4_ and TOF_4/16/9.5_. **b** Correlation was negligible (*r* < 0.3) in lesions with MTV ≤ 15 ml for TOF_4/16/2_, while the threshold was lower for TOF_4/16/6.4_ (MTV ≤ 2.5 ml) and TOF_4/16/9.5_ (MTV ≤ 5.0 ml). The generally lower correlation of ASP and MTV in smaller lesions results from the limited spatial resolution. With TOF_4/16/2_, high noise level contributes to the high MTV threshold for correlation. With TOF_4/16/9.5_, the poorer reconstructed spatial resolution may contribute to the higher MTV threshold compared to TOF_4/16/6.4_

Correlation of ASP and MTV (Fig. 4) for the total patient sample was moderate for TOF_4/16/2_ (Pearson *r* = 0.54; *p* < 0.001) and moderate to high for TOF_4/16/6.4_ (Pearson *r* = 0.69; *p* < 0.001) and TOF_4/16/9.5_ (Pearson *r* = 0.71; *p* < 0.001).

The MTV threshold below which the correlation was negligible (i.e., *r* < 0.3) was highest for TOF_4/16/2_ (MTV ≤ 15 ml) and lowest for TOF_4/16/6.4_ (MTV ≤ 2.5 ml), while it was 5.0 ml for TOF_4/16/9.5_.

## Discussion

This study found that ASP differences between reconstruction algorithms were significantly higher than corresponding SUVmax and MTV differences (Table 2). This may be explained by a combined effect of changes in SUVmax (suppression of local maxima and therefore a decreasing absolute threshold and increasing MTV size) and changes in MTV surface (smoothed, smaller MTV surface) on the ASP. Coarseness of the MTV surface is likely to differ with variation in reconstructed spatial resolution, which—in conventional iterative reconstruction algorithms—is mainly determined by the width of the in-plane filter. Therefore, if threshold-based MTV delineation is applied, wider filters can be expected to result in lower ASP. In Bayesian-penalized likelihood reconstruction (e.g., GE’s Q.Clear), post-processing is not applied, and smoother images are generated by increasing the penalization factor β.

However, since ASP is supposed to serve as part of prognostic/predictive models based on a predefined cutoff, even substantial inter-method differences may be clinically irrelevant if classification of individual patients into groups of high versus low ASP remains concordant. Applying a strict cutoff for ASP of > 19.5% [15], discordantly classified cases compared to the reference algorithm accounted for 2% (TOF_4/8_) or 4% (PSF + TOF_2/17_) at spatial resolution of approx. 7-mm FWHM. This could be acknowledged as acceptably low for application of ASP in a multicenter study. If a less strict cutoff with ± 5% tolerance (ASP between 18.53% and 20.48%) was applied, no discordant cases at 7-mm FWHM were observed for TOF_4/8_ and PSF + TOF_2/17_. This underlines that inter-method ASP differences at comparable spatial resolution are clinically relevant only if ASP is close to the predefined cutoff. Furthermore, this range of tolerance is well covered by the range of possible ASP cutoffs (17% to 39%) within which ASP remained significantly prognostic for PFS in previously reported patients with UICC stage II NSCLC [15].

Relative differences and discordant proportions tended to be higher with Q.Clear. Notably, Q.Clear showed systematically lower image noise at any level of spatial resolution (Table 1 and Fig. 2). In contrast to conventional algorithms, relative ASP differences with Q.Clear compared to the reference algorithm were higher at 7 versus 7 mm than at 5 versus 7 mm (Table 2) or at 7 versus 9 mm (Additional file 2: Table S8). Simultaneously, noise levels at 5 versus 7 mm and 7 versus 9 mm were also more comparable to the reference algorithm than at 7 versus 7 mm. However, the same observation was not true for SUVmax and MTV or with the conventional algorithms. Consequently, similar reconstructed spatial resolution rather than the noise level should guide the choice of reconstruction algorithms for harmonization for multicenter purposes. Furthermore, Q.Clear, or Bayesian-penalized likelihood reconstruction in general, may not be optimal to achieve minimal ASP deviations if the reference is a conventional algorithm.

With the PET scanner used in the present study, variation of image noise between algorithms was especially prominent at spatial resolution of 5-mm FWHM (Table 1, Fig. 1). This partly explains high inter-method differences, which exceeded 100% for TOF_4/8_ and TOF_4/16_ (Table 2), and frequent discordant cases even if pairs of algorithms with 5 versus 5 mm FWHM were compared. In addition to higher noise, Gibbs artifacts (edge elevations) caused by PSF + TOF and Q.Clear reconstruction increase with narrower in-plane filters or lower β [40]. Consequently, SUVmax differences will be more prominent than at 7 mm or 9 mm FWHM. In contrast, in substantially smoothed data with 9-mm FWHM, PET parameters that are reflective of heterogeneity or irregularity of tracer accumulation, such as ASP may lose discriminatory power to detect “real” and clinically relevant differences between tumors/patients. Therefore, under the conditions of the current analysis, 7-mm FWHM could be a feasible and reasonable target for harmonization in a multicenter approach. This is underlined by the observation that the MTV threshold for correlation between ASP and MTV was lowest for TOF_4/16/6.4_ compared to TOF_4/16/9.5_ and especially TOF_4/16/2_.

If reconstructed spatial resolution is better than the target resolution (e.g., 5 mm instead of 7-mm FWHM), retrospective smoothing of data using formula *(**1**)* can be performed to achieve the anticipated resolution. This enabled inter-method differences and discordant proportions far closer to those observed with the original 7-mm data, irrespective of TOF, PSF + TOF or Q.Clear. Consequently, in a multicenter analysis, retrospective smoothing of data with better spatial resolution would be a valid option to ensure comparability. It is important to note that here the effective reconstructed spatial resolution is relevant [28], which can differ notably from the resolution determined via point sources.

A similar approach by the EANM Research Ltd. (EARL) harmonization project was reported by Kaalep et al*.* who analyzed SUV and MTV in FDG-PET data of NSCLC and lymphoma patients. Only after applying an additional Gaussian post-reconstruction filter of 6- to 7-mm FWHM to PET data reconstructed with PSF + TOF (compliant with the current EARL 2 standard) could SUV and MTV differences be reduced from approx. 30% to < 10% compared to reconstruction compliant with the former EARL 1 standard [41]. In a different approach to harmonization, Tsutsui et al*.* examined OSEM + TOF data of a NEMA IEC phantom obtained with a Siemens Biograph mCT and showed that errors compared to a simulated reference phantom were lowest with an in-plane filter of approx. 7- to 8-mm FWHM [42]. In a different study, the group achieved harmonization between 12 different PET scanners using contrast recovery (CR) of NEMA IEC phantom spheres by applying a scanner-specific Gaussian filter of up to 8-mm FWHM [43]. The current results of low SUVmax differences < 5% and MTV differences ≤ 6% at 7 versus 7 mm FWHM imply that both CR and reconstructed spatial resolution may be suitable surrogates for harmonization.

Shorter acquisition times of 120 s, 90 s or 60 s increased inter-method differences compared to 180 s with TOF_4/8/6_ and TOF_4/16/6.4_, while the increase was insignificant or less prominent with PSF + TOF_2/17/7_ and Q.Clear_1750_. More importantly, proportions of discordantly classified cases by ASP, SUVmax or MTV remained similar or did not increase significantly—especially between 180 and 90 s. Therefore, equal acquisition times between PET systems/centers may be of secondary importance to achieve comparability in the investigated parameters, and differences as high as 180 s versus 90 s might be tolerable.

Voxel sizes may also vary between PET systems in a multicenter study. However, due to technical restrictions voxel size could not be freely varied during image reconstruction in this study. Therefore, the influence on ASP, SUVmax and MTV and the correcting effect of retrospective reslicing to the original voxel size could not be assessed. A further limitation of the current analysis is that the variation in reconstruction algorithms and acquisition time may not fully reflect differences between PET scanners beyond these factors. This would require comparative examinations with different scanners in each patient under identical conditions [20, 44]. For methodological consistency with the previous studies [13–15], the same threshold-based algorithm [29] was used to delineate all lesions. Consequently, the presented results are not necessarily valid when lesions are delineated differently. Furthermore, although the current study demonstrated that the reconstructed spatial resolution can be used as a surrogate for scanner harmonization and showed lowest inter-method ASP differences and the lowest MTV threshold for correlation between ASP and MTV for 7.0 FWHM, this is not sufficient for a general recommendation of this specific spatial resolution for future studies regarding the ASP. This decision should also consider the performance of all PET scanners used in a specific study (best achievable reconstructed spatial resolution) and—if available—comparative clinical results on the value of ASP at different reconstructed spatial resolution.

## Conclusions

Differences in ASP, SUVmax and MTV resulting from TOF_4/8_, PSF + TOF_2/17_ or Q.Clear compared to the reference algorithm TOF_4/16_ were mainly determined by differences in reconstructed spatial resolution. Therefore, harmonization for ASP in multicenter studies should aim at comparable reconstructed spatial resolution between PET systems, which is determined by either in-plane filter width or the penalization factor β. With the PET scanner used in the present study, a resolution of 7-mm FWHM ensured that discordantly classified cases of high versus low ASP were at an acceptable proportion for TOF and PSF + TOF of < 5% (Q.Clear: 10%). Retrospectively smoothing data with better spatial resolution (i.e., lower FWHM) to the desired FWHM resulted in comparable results. These results require confirmation in a multicenter study.

## Supplementary information

**Additional file 1**. IBSI Checklist version 1.0 (October 2019; see reference below).

**Additional file 2: Table S1**. Discordant cases relative to the reference algorithm (SUVmax and MTV).** Table S2**. Relative differences to the reference algorithm: Acquisition times (ASP). **Table S3**. Relative differences to the reference algorithm: Acquisition times (SUVmax). **Table S4**. Relative differences to the reference algorithm: Acquisition times (MTV). **Table S5**. Discordant cases relative to the reference algorithm: Acquisition times (ASP). **Table S6**. Discordant cases relative to the reference algorithm: Acquisition times (SUVmax). **Table S7**. Discordant cases relative to the reference algorithm: Acquisition times (MTV). **Table S8**. Relative differences and discordant cases relative to the reference algorithm (7 vs. 9 mm FWHM).

## Data Availability

The datasets used and/or analyzed during the current study are available from the corresponding author on reasonable request.

## Abbreviations

95% CI: 95% Confidence interval
ASP: Asphericity
CR: Contrast recovery
EANM: European Association of Nuclear Medicine
EARL: EANM Research Ltd.
FDG: Fluorodeoxyglucose
FWHM: Full width at half maximum
IBSI: Image Biomarker Standardization Initiative
IEC: International Electrotechnical Commission
it: Iterations
IQR: Interquartile range
MTV: Metabolic tumor volume
NEMA: National Electrical Manufacturers Association
NSCLC: Non-small cell lung cancer
OS: Overall survival
OSEM: Ordered subset expectation maximization
PET/CT: Positron emission tomography/computed tomography
PFS: Progression-free survival
PSF: Point spread function
SiPM: Silicon photomultipliers
ss: Subsets
SUV: Standardized uptake value
TOF: Time of flight
VOI: Volume of interest
