# Supplementary material for: Asphericity of tumor FDG uptake in non-small cell lung cancer: reproducibility and implications for harmonization in multicenter studies
Source: EJNMMI Res. 2020 Nov 2;10:134. doi: 10.1186/s13550-020-00725-y (PMC7606415; doi:10.1186/s13550-020-00725-y)
Supplement: Supplementary file 1 — Additional file 1. IBSI Checklist version 1.0 (October 2019; see reference below). [file 13550_2020_725_MOESM1_ESM.docx]

**Additional file 1**

IBSI Checklist version 1.0 (October 2019; *see reference below*)

| **Topic** |  | **Item** | **Description** | **Page** |
| --- | --- | --- | --- | --- |
| **Patient** |  |  |  |  |
| Region of interest^12^ |  | 1 | Describe the region of interest that is being imaged. | 7 |
| Patient preparation |  | 2a | Describe specific instructions given to patients prior to image acquisition, eg, fasting prior to imaging. | 7 |
|  |  | 2b | Describe administration of drugs to the patient prior to image acquisition, eg, muscle relaxants. | none |
|  |  | 2c | Describe the use of specific equipment for patient comfort during scanning, eg, ear plugs. | none |
| Radioactive tracer | PET, SPECT | 3a | Describe which radioactive tracer was administered to the patient, eg, 18F-FDG. | 7 |
|  | PET, SPECT | 3b | Describe the administration method. | 7 |
|  | PET, SPECT | 3c | Describe the injected activity of the radioactive tracer at administration. | 7 |
|  | PET, SPECT | 3d | Describe the uptake time prior to image acquisition. | 7 |
|  | PET, SPECT | 3e | Describe how competing substance levels were controlled.^13^ | n.a. |

| **Acquisition^15^** |  |  |  |  |
| --- | --- | --- | --- | --- |
| Acquisition protocol |  | 6 | Describe whether a standard imaging protocol was used, and where its description may be found. | none |
| Scanner type |  | 7 | Describe the scanner type (s) and vendor (s) used in the study. | 6 and 7 |
| Imaging modality |  | 8 | Clearly state the imaging modality that was used in the study, eg, CT, MRI. | 7 |
| Static/dynamic scans |  | 9a | State if the scans were static or dynamic. | 7 |
| Scanner calibration |  | 10 | Describe how and when the scanner was calibrated. | 6 |
| Patient instructions |  | 11 | Describe specific instructions given to the patient during acquisition, eg, breath holding. | none |
| Anatomic motion correction |  | 12 | Describe the method used to minimize the effect of anatomic motion. | none |
| Scan duration |  | 13 | Describe the duration of the complete scan or the time per bed position. | 7 |
| Time-of-flight | PET | 16 | State if scanner time-of-flight capabilities are used during acquisition. | 6 |

| **Reconstruction^16^** |  |  |  |  |
| --- | --- | --- | --- | --- |
| In-plane resolution |  | 28 | Describe the distance between pixels, or alternatively the field of view and matrix size. | 6 |
| Image slice thickness |  | 29 | Describe the slice thickness. | 6 |
| Image slice spacing |  | 30 | Describe the distance between image slices.^17^ | n.a. |
| Reconstruction method | PET | 32a | Describe which reconstruction method was used, eg, 3D OSEM. | 6 |
|  | PET | 32b | Describe the number of iterations for iterative reconstruction. | 6 |
|  | PET | 32c | Describe the number of subsets for iterative reconstruction. | 6 |
| Point spread function modeling | PET | 33 | Describe if and how point-spread function modeling was performed. | 6 |
| Image corrections | PET | 34a | Describe if and how attenuation correction was performed. | 6 and 7 |
|  | PET | 34b | Describe if and how other forms of correction were performed, eg, scatter correction, randoms correction, dead time correction etc | 6 |

| **Image processing-data conversion** | | | |  |
| --- | --- | --- | --- | --- |
| SUV normalization | PET | 38 | Describe which standardized uptake value (SUV) normalization method is used. | 8 |
| Other data conversions |  | 40 | Describe any other conversions that are performed to generate eg, perfusion maps. | 7 |

| **Image processing-postacquisition processing** | | | |  |
| --- | --- | --- | --- | --- |
| Antialiasing |  | 41 | Describe the method used to deal with antialiasing when downsampling during interpolation. | n.a. |
| Noise suppression |  | 42 | Describe methods used to suppress image noise. | none |
| Postreconstruction smoothing filter | PET | 43 | Describe the width of the Gaussian filter (FWHM) to spatially smooth intensities. | 8 |
| Intensity normalization |  | 46 | Describe the method and settings used to normalize intensity distributions within a patient or patient cohort. | none |
| Other postacquisition processing methods |  | 47 | Describe any other methods that were used to process the image and are not mentioned separately in this list. | none |

| **Segmentation** |  |  |  |  |
| --- | --- | --- | --- | --- |
| Segmentation method |  | 48a | Describe how regions of interest were segmented, eg, manually. | 8 |
|  |  | 48b | Describe the number of experts, their expertise and consensus strategies for manual delineation. | 8 |
|  |  | 48c | Describe methods and settings used for semiautomatic and fully automatic segmentation. | 8 |
|  |  | 48d | Describe which image was used to define segmentation in case of multimodality imaging. | 8 |
| Conversion to mask |  | 49 | Describe the method used to convert polygonal or mesh-based segmentations to a voxel-based mask. | n.a. |

| **Image processing-image interpolation** | | | |  |
| --- | --- | --- | --- | --- |
| Interpolation method |  | 50a | Describe which interpolation algorithm was used to interpolate the image. | n.a. |
|  |  | 50b | Describe how the position of the interpolation grid was defined, eg, align by center. | n.a. |
|  |  | 50c | Describe how the dimensions of the interpolation grid were defined, eg, rounded to nearest integer. | n.a. |
|  |  | 50d | Describe how extrapolation beyond the original image was handled. | n.a. |
| Voxel dimensions |  | 51 | Describe the size of the interpolated voxels. | n.a. |

| **Image processing-ROI interpolation** | | | |  |
| --- | --- | --- | --- | --- |
| Interpolation method |  | 53 | Describe which interpolation algorithm was used to interpolate the region of interest mask. | n.a. |
| Partially masked voxels |  | 54 | Describe how partially masked voxels after interpolation are handled. | n.a. |

| **Image processing-resegmentation** | | | |  |
| --- | --- | --- | --- | --- |
| Resegmentation methods |  | 55 | Describe which methods and settings are used to resegment the ROI intensity mask. | n.a. |

| **Image processing-discretization** | | | |  |
| --- | --- | --- | --- | --- |
| Discretization method^19^ |  | 56a | Describe the method used to discretise image intensities. | n.a. |
|  |  | 56b | Describe the number of bins (FBN) or the bin size (FBS) used for discretization. | n.a. |
|  |  | 56c | Describe the lowest intensity in the first bin for FBS discretization.^20^ | n.a. |

| **Image processing-image transformation** | | | |  |
| --- | --- | --- | --- | --- |
| Image filter^21^ |  | 57 | Describe the methods and settings used to filter images, eg, Laplacian-of-Gaussian. | n.a. |

| **Radiomics feature computation** | | | |  |
| --- | --- | --- | --- | --- |
| Feature set |  | 58 | Describe which set of radiomics features is computed and refer to their definitions or provide these. | 8 |
| IBSI compliance |  | 59 | State if the software used to extract the set of features is able to reproduce the IBSI feature reference values.^22^ | 9 |
| Robustness |  | 60 | Describe how robustness of the features was assessed, eg, testretest analysis. | n.a. |
| Software availability |  | 61 | Describe which software and version was used to compute features. | 8 |

Reference for this IBS checklist:

Zwanenburg A, Vallières M, Abdalah MA *et al.* The Image Biomarker Standardization Initiative: Standardized Quantitative Radiomics for High-Throughput Image-based Phenotyping. Radiology. 295(2):191145
