# Supplementary material for: Asphericity of tumor FDG uptake in non-small cell lung cancer: reproducibility and implications for harmonization in multicenter studies
Source: EJNMMI Res. 2020 Nov 2;10:134. doi: 10.1186/s13550-020-00725-y (PMC7606415; doi:10.1186/s13550-020-00725-y)
Supplement: Supplementary file 2 — Additional file 2: Table S1. Discordant cases relative to the reference algorithm (SUVmax and MTV). Table S2. Relative differences to the reference algorithm: Acquisition times (ASP). Table S3. Relative differences to the reference algorithm: Acquisition times (SUVmax). Table S4. Relative differences to the reference algorithm: Acquisition times (MTV). Table S5. Discordant cases relative to the reference algorithm: Acquisition times (ASP). Table S6. Discordant cases relative to the reference algorithm: Acquisition times (SUVmax). Table S7. Discordant cases relative to the reference algorithm: Acquisition times (MTV). Table S8. Relative differences and discordant cases relative to the reference algorithm (7 vs. 9 mm FWHM). [file 13550_2020_725_MOESM2_ESM.docx]

**Additional file 2**

**Table S1.** Discordant cases relative to the reference algorithm (SUVmax and MTV)

|  | **Discordant proportion, % (95%-CI)** | | | | |
| --- | --- | --- | --- | --- | --- |
|  | **5 mm vs. 7 mm** | **7 mm vs. 7 mm** | **9 mm vs. 7 mm** | **5 mm vs. 5 mm** | **9 mm vs. 9 mm** |
| **SUVmax (10.5)** |  |  |  |  |  |
| TOF_4/8_ | 22 (9.5 to 34.5) | 6 (0 to 13.6) | 10 (0.7 to 19.3) | 6 (0 to 13.6) | 0 (0 to 1.0) |
| TOF_4/16_ | 28 (14.6 to 41.4) | --- | 10 (0.7 to 19.3) | --- | --- |
| PSF+TOF_2/17_ | 22 (9.5 to 34.5) | 4 (0 to 10.4) | 8 (0 to 16.5) | 6 (0 to 13.6) | 2 (0 to 6.9) |
| Q.Clear | 16 (4.8 to 27.2) | 8 (0 to 16.5) | 10 (0.7 to 19.3) | 12 (2.0 to 22.0) | 4 (0 to 10.4) |
| **MTV (9.5 ml)** |  |  |  |  |  |
| TOF_4/8_ | 20 (7.9 to 32.1) | 2 (0 to 6.9) | 2 (0 to 6.9) | 0 (0 to 1.0) | 0 (0 to 1.0) |
| TOF_4/16_ | 20 (7.9 to 32.1) | --- | 2 (0 to 6.9) | --- | --- |
| PSF+TOF_2/17_ | 22 (9.5 to 34.5) | 4 (0 to 10.4) | 2 (0 to 6.9) | 6 (0 to 13.6) | 0 (0 to 1.0) |
| Q.Clear | 8 (0 to 16.5) | 8 (0 to 16.5) | 8 (0 to 16.5) | 16 (4.8 to 27.2) | 10 (0.7 to 19.3) |

Proportions of discordantly classified cases among all 50 patients are given in % (95%-confidence interval; 95%-CI) for each algorithm relative to the reference algorithm TOF_4/16_. Different pairs of reconstructed spatial resolution (FWHM) are compared. Missing values reflect pairs of identical datasets.

**Table S2.** Relative differences to the reference algorithm: Acquisition times (ASP)

| **ASP** | **Difference, % (median, IQR)** |  | **p value** |  |
| --- | --- | --- | --- | --- |
|  |  | **180s vs. 120s** | **180s vs. 90s** | **180s vs. 60s** |
| TOF_4/8_ 180s | 7.6 (3.1 to 18.0) |  |  |  |
| 120s | 9.9 (3.2 to 34.6) | **0.007** |  |  |
| 90s | 12.3 (4.9 to 34.7) |  | **0.007** |  |
| 60s | 23.4 (0.9 to 40.4) |  |  | **<0.001** |
| TOF_4/16_ 180s | --- |  |  |  |
| 120s | 7.1 (3.5 to 18.4) | **<0.001** |  |  |
| 90s | 15.0 (2.8 to 35.3) |  | **<0.001** |  |
| 60s | 27.0 (9.3 to 54.7) |  |  | **<0.001** |
| PSF+TOF_2/17_ 180s | 12.8 (6.3 to 26.9) |  |  |  |
| 120s | 11.0 (6.7 to 24.1) | 0.68 |  |  |
| 90s | 13.2 (4.3 to 24.0) |  | 0.43 |  |
| 60s | 13.4 (6.0 to 44.1) |  |  | 0.79 |
| Q.Clear 180s | 31.3 (11.2 to 43.7) |  |  |  |
| 120s | 24.1 (8.2 to 39.0) | 0.12 |  |  |
| 90s | 24.2 (10.1 to 36.1) |  | 0.26 |  |
| 60s | 14.2 (3.3 to 31.6) |  |  | **0.008** |

Relative differences in % (with interquartile range; IQR) are given for each algorithm at different acquisition times relative to the reference algorithm TOF_4/16_ at 7 mm FWHM (i.e., TOF_4/16/6.4_). Acquisition time of the reference was always 180s. Results of the Wilcoxon test comparing relative differences at 180s with differences at 120s, 90s or 60s are given (significant p values printed in bold). Missing values reflect pairs of identical datasets.

**Table S3.** Relative differences to the reference algorithm: Acquisition times (SUVmax)

| **SUVmax** | **Difference, % (median, IQR)** |  | **p value** |  |
| --- | --- | --- | --- | --- |
|  |  | **180s vs. 120s** | **180s vs. 90s** | **180s vs. 60s** |
| TOF_4/8_ 180s | 1.2 (0.6 to 2.0) |  |  |  |
| 120s | 1.9 (0.9 to 3.2) | **0.012** |  |  |
| 90s | 2.9 (1.5 to 4.7) |  | **<0.001** |  |
| 60s | 4.0 (2.1 to 7.4) |  |  | **<0.001** |
| TOF_4/16_ 180s | --- |  |  |  |
| 120s | 2.2 (1.1 to 3.8) | **<0.001** |  |  |
| 90s | 2.3 (1.0 to 5.3) |  | **<0.001** |  |
| 60s | 3.7 (1.5 to 7.5) |  |  | **<0.001** |
| PSF+TOF_2/17_ 180s | 4.9 (1.9 to 7.2) |  |  |  |
| 120s | 4.8 (2.3 to 7.9) | 0.21 |  |  |
| 90s | 5.6 (3.4 to 8.6) |  | **0.027** |  |
| 60s | 6.5 (3.9 to 10.6) |  |  | **0.006** |
| Q.Clear 180s | 5.1 (2.4 to 10.6) |  |  |  |
| 120s | 6.1 (2.0 to 9.5) | 0.81 |  |  |
| 90s | 5.4 (3.0 to 8.4) |  | 0.72 |  |
| 60s | 5.0 (3.4 to 9.3) |  |  | 0.31 |

Relative differences in % (with interquartile range; IQR) are given for each algorithm at different acquisition times relative to the reference algorithm TOF_4/16_ at 7 mm FWHM (i.e., TOF_4/16/6.4_). Acquisition time of the reference was always 180s. Results of the Wilcoxon test comparing relative differences at 180s with differences at 120s, 90s or 60s are given (significant p values printed in bold). Missing values reflect pairs of identical datasets.

**Table S4.** Relative differences to the reference algorithm: Acquisition times (MTV)

| **MTV** | **Difference, % (median, IQR)** |  | **p value** |  |
| --- | --- | --- | --- | --- |
|  |  | **180s vs. 120s** | **180s vs. 90s** | **180s vs. 60s** |
| TOF_4/8_ 180s | 2.3 (1.4 to 4.4) |  |  |  |
| 120s | 3.6 (1.7 to 5.4) | **0.028** |  |  |
| 90s | 4.9 (2.4 to 7.2) |  | **0.001** |  |
| 60s | 6.7 (3.8 to 11.2) |  |  | **<0.001** |
| TOF_4/16_ 180s | --- |  |  |  |
| 120s | 3.3 (1.6 to 5.1) | **<0.001** |  |  |
| 90s | 3.8 (1.6 to 8.2) |  | **<0.001** |  |
| 60s | 7.1 (2.1 to 10.6) |  |  | **<0.001** |
| PSF+TOF_2/17_ 180s | 6.1 (3.1 to 12.7) |  |  |  |
| 120s | 6.6 (2.6 to 13.7) | 0.85 |  |  |
| 90s | 7.3 (3.9 to 11.0) |  | 0.55 |  |
| 60s | 10.2 (6.1 to 15.1) |  |  | **0.041** |
| Q.Clear 180s | 6.3 (3.1 to 13.2) |  |  |  |
| 120s | 8.4 (3.3 to 14.1) | 0.39 |  |  |
| 90s | 6.6 (3.6 to 10.7) |  | 0.73 |  |
| 60s | 7.5 (3.6 to 13.1) |  |  | 0.36 |

Relative differences in % (with interquartile range; IQR) are given for each algorithm at different acquisition times relative to the reference algorithm TOF_4/16_ at 7 mm FWHM (i.e., TOF_4/16/6.4_). Acquisition time of the reference was always 180s. Results of the Wilcoxon test comparing relative differences at 180s with differences at 120s, 90s or 60s are given (significant p values printed in bold). Missing values reflect pairs of identical datasets.

**Table S5.** Discordant cases relative to the reference algorithm: Acquisition times (ASP)

| **ASP** | **Proportion, % (95%-CI)** |  | **p value** |  |
| --- | --- | --- | --- | --- |
|  |  | **180s vs. 120s** | **180s vs. 90s** | **180s vs. 60s** |
| TOF_4/8_ 180s | 2 (0 to 6.9) |  |  |  |
| 120s | 2 (0 to 6.9) | 1.0 |  |  |
| 90s | 2 (0 to 6.9) |  | 1.0 |  |
| 60s | 8 (0 to 16.5) |  |  | 0.38 |
| TOF_4/16_ 180s | --- |  |  |  |
| 120s | 4 (0 to 10.4) | --- |  |  |
| 90s | 2 (0 to 6.9) |  | --- |  |
| 60s | 14 (3.4 to 24.6) |  |  | --- |
| PSF+TOF_2/17_ 180s | 4 (0 to 10.4) |  |  |  |
| 120s | 4 (0 to 10.4) | 1.0 |  |  |
| 90s | 2 (0 to 6.9) |  | 1.0 |  |
| 60s | 4 (0 to 10.4) |  |  | 1.0 |
| Q.Clear 180s | 10 (0.7 to 19.3) |  |  |  |
| 120s | 8 (0 to 16.5) | 1.0 |  |  |
| 90s | 10 (0.7 to 19.3) |  | 1.0 |  |
| 60s | 10 (0.7 to 19.3) |  |  | 1.0 |

Proportions of discordantly classified cases among all 50 patients are given in % (95%-confidence interval; 95%-CI) for each algorithm at different acquisition times relative to the reference algorithm TOF_4/16_ at 7 mm FWHM (i.e., TOF_4/16/6.4_). Acquisition time of the reference was always 180s. Results of McNemar’s test comparing discordant proportions at 180s with differences at 120s, 90s or 60s are given (significant p values printed in bold). Missing values reflect pairs of identical datasets or pairs where McNemar’s test cannot be performed.

**Table S6.** Discordant cases relative to the reference algorithm: Acquisition times (SUVmax)

| **SUVmax** | **Proportion, % (95%-CI)** |  | **p value** |  |
| --- | --- | --- | --- | --- |
|  |  | **180s vs. 120s** | **180s vs. 90s** | **180s vs. 60s** |
| TOF_4/8_ 180s | 6 (0 to 13.6) |  |  |  |
| 120s | 6 (0 to 13.6) | 1.0 |  |  |
| 90s | 6 (0 to 13.6) |  | 1.0 |  |
| 60s | 6 (0 to 13.6) |  |  | 1.0 |
| TOF_4/16_ 180s | --- |  |  |  |
| 120s | 4 (0 to 10.4) | --- |  |  |
| 90s | 4 (0 to 10.4) |  | --- |  |
| 60s | 4 (0 to 10.4) |  |  | --- |
| PSF+TOF_2/17_ 180s | 4 (0 to 10.4) |  |  |  |
| 120s | 8 (0 to 16.5) | 0.5 |  |  |
| 90s | 6 (0 to 13.6) |  | 1.0 |  |
| 60s | 8 (0 to 16.5) |  |  | 0.5 |
| Q.Clear 180s | 8 (0 to 16.5) |  |  |  |
| 120s | 8 (0 to 16.5) | 1.0 |  |  |
| 90s | 6 (0 to 13.6) |  | 1.0 |  |
| 60s | 6 (0 to 13.6) |  |  | 1.0 |

Proportions of discordantly classified cases among all 50 patients are given in % (95%-confidence interval; 95%-CI) for each algorithm at different acquisition times relative to the reference algorithm TOF_4/16_ at 7 mm FWHM (i.e., TOF_4/16/6.4_). Acquisition time of the reference was always 180s. Results of McNemar’s test comparing discordant proportions at 180s with differences at 120s, 90s or 60s are given (significant p values printed in bold). Missing values reflect pairs of identical datasets or pairs where McNemar’s test cannot be performed.

**Table S7.** Discordant cases relative to the reference algorithm: Acquisition times (MTV)

| **MTV** | **Proportion, % (95%-CI)** |  | **p value** |  |
| --- | --- | --- | --- | --- |
|  |  | **180s vs. 120s** | **180s vs. 90s** | **180s vs. 60s** |
| TOF_4/8_ 180s | 2 (0 to 6.9) |  |  |  |
| 120s | 4 (0 to 10.4) | 1.0 |  |  |
| 90s | 6 (0 to 13.6) |  | 0.5 |  |
| 60s | 10 (0.7 to 19.3) |  |  | 0.13 |
| TOF_4/16_ 180s | --- |  |  |  |
| 120s | 2 (0 to 6.9) | --- |  |  |
| 90s | 4 (0 to 10.4) |  | --- |  |
| 60s | 10 (0.7 to 19.3) |  |  | --- |
| PSF+TOF_2/17_ 180s | 4 (0 to 10.4) |  |  |  |
| 120s | 6 (0 to 13.6) | 1.0 |  |  |
| 90s | 12 (2.0 to 22.0) |  | 0.13 |  |
| 60s | 10 (0.7 to 19.3) |  |  | 0.25 |
| Q.Clear 180s | 8 (0 to 16.5) |  |  |  |
| 120s | 8 (0 to 16.5) | 1.0 |  |  |
| 90s | 8 (0 to 16.5) |  | 1.0 |  |
| 60s | 8 (0 to 16.5) |  |  | 1.0 |

Proportions of discordantly classified cases among all 50 patients are given in % (95%-confidence interval; 95%-CI) for each algorithm at different acquisition times relative to the reference algorithm TOF_4/16_ at 7 mm FWHM (i.e., TOF_4/16/6.4_). Acquisition time of the reference was always 180s. Results of McNemar’s test comparing discordant proportions at 180s with differences at 120s, 90s or 60s are given (significant p values printed in bold). Missing values reflect pairs of identical datasets or pairs where McNemar’s test cannot be performed.

**Table S8.** Relative differences and discordant cases relative to the reference algorithm (7 vs. 9 mm FWHM)

|  | **Difference, % (median, IQR)** | **Discordant proportion, % (95%-CI)** |
| --- | --- | --- |
|  | **7 mm vs. 9 mm** | **7 mm vs. 9 mm** |
| **ASP (19.5%)** |  |  |
| TOF_4/8/6_ | 46.5 (25.6 to 81.5) | 12 (2.0 to 22.0) |
| TOF_4/16/6.4_ | 46.3 (30.3 to 88.3) | 14 (3.4 to 24.6) |
| PSF+TOF_2/17/7_ | 37.6 (22.7 to 66.3) | 14 (3.4 to 24.6) |
| Q.Clear_1750_ | 23.8 (14.0 to 63.3) | 12 (2.0 to 22.0) |
| **SUVmax (10.5)** |  |  |
| TOF_4/8/6_ | 14.5 (11.3 to 22.4) | 12 (2.0 to 22.0) |
| TOF_4/16/6.4_ | 14.5 (10.9 to 19.7) | 10 (0.7 to 19.3) |
| PSF+TOF_2/17/7_ | 18.4 (14.1 to 26.8) | 14 (3.4 to 24.6) |
| Q.Clear_1750_ | 18.5 (11.7 to 25.8) | 18 (6.4 to 29.6) |
| **MTV (9.5 ml)** |  |  |
| TOF_4/8/6_ | 13.7 (6.6 to 23.0) | 4 (0 to 10.4) |
| TOF_4/16/6.4_ | 11.0 (6.0 to 18.3) | 2 (0 to 6.9) |
| PSF+TOF_2/17/7_ | 17.7 (9.1 to 27.1) | 6 (0 to 13.6) |
| Q.Clear_1750_ | 17.0 (10.4 to 27.4) | 10 (0.7 to 19.3) |

Relative differences in % (with interquartile range; IQR) proportions of discordantly classified cases (%; 95%-confidence interval; 95%-CI) are given for each algorithm at 7.0 FWHM compared to the reference algorithm TOF_4/16_ at 9 mm FWHM (i.e., TOF_4/16/9.5_). The respective cut-off values to define positive cases are provided for ASP, SUVmax and MTV in parentheses.
